# Antibiofilm Activity of Biocide Metal Ions Containing Bioactive Glasses (BGs): A Mini Review

**DOI:** 10.3390/bioengineering9100489

**Published:** 2022-09-21

**Authors:** Irina Atkinson

**Affiliations:** “Ilie Murgulescu” Institute of Physical Chemistry of the Romanian Academy, 202, Spl. Independentei, 060021 Bucharest, Romania; irinaatkinson@yahoo.com

**Keywords:** bacterial biofilm, bioactive glass, metal ions

## Abstract

One of the major clinical issues during the implantation procedure is the bacterial infections linked to biofilms. Due to their tissue localization and the type of bacteria involved, bacterial infections at implant sites are usually difficult to treat, which increases patient morbidity and even mortality. The difficulty of treating biofilm-associated infections and the emergence of multidrug-resistant bacteria are further challenges for the scientific community to develop novel biomaterials with excellent biocompatibility and antibacterial properties. Given their ability to stimulate bone formation and have antibacterial properties, metal ion-doped bioactive glasses (BGs) have received considerable research. This mini review aims to be successful in presenting the developments made about the role of biocide metal ions incorporated into BGs against the development of bacterial biofilms and the spread of nosocomial diseases.

## 1. Introduction

One of the most challenging issues in the healthcare industry is the occurrence of antibiotic-resistant bacterial infections. The World Health Organization (WHO) has ranked bacterial infections among its top 10 research priorities. Worldwide, the mortality associated with antibiotic-resistant infections is estimated at 7 million deaths per year, and it is anticipated to increase to 10 million deaths per year in 2050 if efficient therapies are not found [1,2]. Furthermore, it is predicted that the cost of treatment related to bacterial infections in Europe will rise by more than EUR 1.5 billion/year as a result of the spread and increase of bacterial diseases [3].

Antibiotics of various types have been developed to control and treat bacterial infections. Penicillin, among the several antibiotics produced in the 1940s, quickly developed resistance as a result of use and even abuse. Moreover, because antibiotics in clinical use concentrate on a limited number of biological targets, drug resistance might develop over time.

In addition to the aforementioned phenomena, a strategy used by bacteria for their survival is grouping into complex surface-attached communities, protected by a self-produced polymer matrix of polysaccharides, produced proteins, and extracellular DNA called biofilms [4,5,6,7]. Compared to their planktonic cells, the bacteria enclosed in the biofilm have better survival options such as access to nutrients, the ability to grow in oligotrophic conditions, resistance to biocides, and environmental stability.

**Figure 1:**
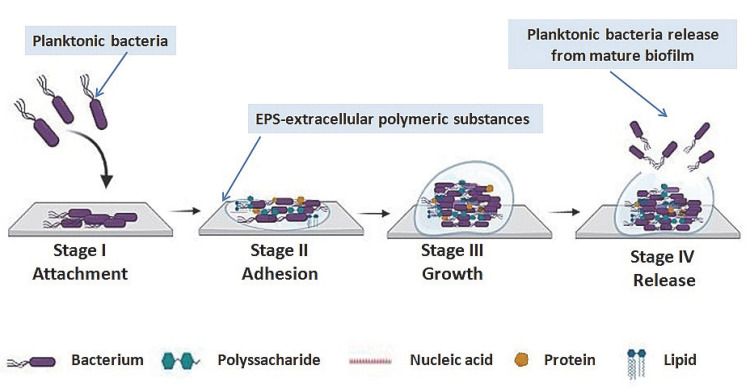
The stages of biofilm formation (created with BioRender.com (accessed on 1 September 2022)).

The development of biofilms occurs in four stages [8,9]: (I) the initial reversible attachment occurs when the bacterial cell interacts with the substrate; (II) irreversible attachment takes place when the bacterial cells connect with the surface by secreting extracellular polymeric substances (EPS) and employing an adhesive-like lipopolysaccharide; (III) as the biofilm develops and reaches maturity, the bacterial cells are encapsulated in an extracellular matrix (ECM) which is formed of proteins, polysaccharides, and extracellular DNA that enables the attachment of other species; (IV) at maturity, the biofilm is capable of releasing part of its bacterial cells, which return to independent planktonic life (Figure 1).

Bacterial biofilm is responsible for more than 60% of nosocomial infections linked to some implanted medical devices [10] and is also associated with chronic infections, i.e., osteomyelitis. The common characteristic of biofilm-associated infections is their inherent resistance to host immunity, antibiotics, and biocides [11]. It is known that sessile bacteria need higher levels of antibiotics compared to minimum inhibitory concentrations of the corresponding planktonic form [12]. A wide range of opportunistic bacteria regularly found within the microflora of the implant site are able to produce biofilm on prosthetic materials, usually causing implant therapy failure. Both types of bacteria, Gram-negative and Gram-positive, can form biofilm on medical devices. The most common bacteria that undergo planktonic to sessile transition are *E. faecalis*, *S. aureus*, *S. epidermidis*, *P. aeruginosa*, *S. viridans*, *E. coli*, *P. mirabilis*, and *K. pneumonia* [13]. Among them, the Gram-positive bacteria, *S. aureus*, coagulase-negative staphylococci, besides Gram-negative bacteria such as *E. coli* and *P. aeruginosa*, are the most commonly involved pathogens in the biofilm formation on the prosthetic implants [14,15,16].

A variety of approaches have been applied to inhibit the formation of harmful biofilms, including physical methods (ultrasound and magnetic fields treatments) [17,18], altering the surface characteristics of the materials (such as their smoothness, wettability, and hydrophilicity) [19], coating prosthetic implants with a particular material [20], biochemical methods using the degradative enzyme [21], and biomaterials as an antibiotic delivery system [22].

Considerable efforts are being devoted to finding and manufacturing biomaterials that have applications in therapies for bone infections [23,24,25,26]. Bioactive glasses (BGs) have become an emerging field for bone pathologies due to their antibacterial properties. However, when metal ions or drugs are incorporated into the BGs structure, their bactericidal properties are improved.

## 2. Bioactive Glasses

BGs are of great interest in the field of medical implants due to their osteoinductive, osteoproductive, osteoconductive, and antimicrobial properties [27]. L. Hench, who revolutionized the field of biomaterials with his invention of 45S5 BG, known as Bioglass^®^ (wt. %: 45SiO_2_-24.5Na_2_O-24.5CaO-6P_2_O_5_), in the early 1970s, classified a bioactive material as one that causes a biological reaction at its interface and promotes a bond to develop between the tissues and the material [28]. He discovered that the composition of 45S5 bioactive glass bonded with the bone through the formation of hydroxyapatite (HAP), an analog to the mineral phase of bones when it was in contact with biological fluids. The Bioglass^®^ composition has received the approval of the US Food and Drug Administration. Currently, it is used for middle ear treatment and periodontal repair and augmentation [29].

Since then, the research on BGs has provided very good results through the conversion of traditional glasses into glasses with added properties that address healthcare needs. BGs can bond to and integrate with the bone tissue without promoting inflammation and toxicity or forming fibrous tissue [30,31].

The main advantage of BGs for tissue engineering applications is their surface reactivity. The reaction products resulting from the interaction between BGs and the physiological fluids lead to the formation of the HAP-like phase, similar to the crystalline HAP of bones. When these glasses are exposed to an aqueous environment, they undergo several surface reactions that have been described as the bioactivity of BGs [32]:

I. Rapid exchange of Ca^2+^ with a proton or hydrate proton;
II. Generation of silanols (Si–OH) at the site of the breakdown of the silica network. Solution interface for BG. In this stage, soluble silica [Si(OH)4] is also produced and released to the bodily fluid;
III. Condensation and repolymerization of the silica-rich layer take place on the BGs’surface. Consumption of Si–OH;
IV. Ca^2+^ and PO_4_^3−^ migrate to the surface and form Ca–PO_4_^3−^ clusters on the top of the SiO_2_-rich layer, and the crystallization of the amorphous CaP takes place;
V. Finally, the hydroxycarbonate apatite layer (HAC) is formed by the incorporation of OH^–^ and CO_3_^2−^ anions from the solution.

Furthermore, the release of ions makes the surrounding environment hostile to microbial development through the generation of osmotic and acid-base imbalance without being dependent on antibiotics and without harming the host tissues.

BGs can be obtained either by the traditional melt-quenching or the modern sol-gel method [33].

Melt-derived BGs are prepared at temperatures higher than 1000 °C, with a procedure analogous to that used to melt common window glasses. The resulting material does not have any porosity at all, and the surface area depends only on the particle size obtained by grinding up the powders. 

In the 1990s, the sol–gel method, which involves the hydrolysis and polymerization of metal hydroxides, alkoxides, and/or inorganic salts, was used for the first time to create BGs [34]. Contrary to the melt-derived BGs, the sol–gel glasses are not prepared at elevated processing temperatures. The surface and structural properties (such as the surface area and porosity) can be finely modulated depending on the composition and synthesis conditions. The BGs obtained by the sol–gel method can have different types of pores, such as nanopores, macropores, or mesopores [35]. At the very end, controlled nanostructured materials can be obtained. Due to their outstanding textural properties, HAP is deposited much faster on the sol–gel BGs than on the melt-derived ones, and the materials exhibit higher bone-bonding rates, together with excellent degradation/resorption properties [36,37,38].

Furthermore, the sol–gel processes can be combined with the supramolecular chemistry of surfactants, resulting in the third-generation class of BGs referred to as ordered mesoporous bioactive glasses (MBGs), with the values of the surface and porosity up to five times higher than those obtained by the sol–gel method [39,40]. The surfactants for preparing MBGs mainly include CTAB, P123 (EO20-PO70-EO20), and F127 (EO106-PO70-EO106) [41]. MBGs exhibit the highest in vitro bioactivity and their ordered mesoporous structure allows for the incorporation of antimicrobial agents, etc., thereby having a huge potential in the therapy of bacteria-associated infection. 

Moreover, BGs can be used to produce three-dimensional scaffolds, which are an advantage in tissue-engineering applications. Thus, a bioactive, biodegradable, and highly porous matrix that could resemble the cancellous bone is obtained. 

The introduction of the sol–gel method opened the research to new types of materials for medical applications. One of the advantages of this method, in comparison with the conventional melting technique, is the possibility of easy chemical doping. Moreover, the traditional melting method’s higher processing temperature causes some components, including P_2_O_5_, to evaporate throughout the process, decreasing the overall bioactive potential of the glass, as well as other properties [42].

The antibacterial property of the BGs is an intriguing characteristic. In some cases, BG compositions have demonstrated antibacterial properties without any addition to their composition of metal ions or antibiotics.

Zhou et al. [43] compared the efficacy of 45S5 and S53P4 (53SiO_2_-23Na_2_O-20%CaO- 4% P_2_O_5_, wt. %) BGs against the biofilm generated by *MRSA* and *V. parvula*. They found that 45S5 BG particles exhibited a higher reduction of the biofilm than the S53P4 BG particles, suggesting the potential of 45S5 BG for eradicating mature biofilm. They emphasized the influence of the BGs particle size on the effectiveness against biofilms. Smaller particles reduced biofilms significantly more than larger particles across the experiment. Other researchers have demonstrated the strong activity of S53P4 BG to reduce the biofilm produced by a wide variety of bacteria, including *S. aureus*, *P. aeruginosa*, *K. pneumoniae*, and *S. epidermidis* [44,45].

Considering that bacteria can evolve defensive mechanisms against antibiotics through mutation and selection, an alternative to treating bone infections is the use of biocide metal ions. Low-bacterial resistance is one of the advantages of these ions.

Moreover, the concentration of the dopant should be determined, because the low dopant concentrations sometimes may not produce the desired properties, and on the contrary, high dopant concentrations may have cytotoxic and carcinogenic effects [46].

Many studies have suggested the positive effect of metal ions loaded on BGs against bacterial biofilm (Table 1). 

## 3. Mechanism of Antibacterial Action of Metal Ions

The antibacterial mechanism of BG implies the ionic dissolution products that result in a slight increase in the medium pH and osmolarity, creating an inhospitable environment for the proliferation of bacteria, but sometimes the pH alone is insufficient to eradicate the bacteria in the solution [55].

Metal ions with bactericidal action can be incorporated into the glass structure in order to improve the antibiofilm activity of the BGs. When the glass is dissolved in the bodily fluid, these ions are slowly released and interact with cells, inducing changes in their metabolism. Various metal ions with antibacterial assets can be included into the glass network, such as copper (Cu^+^ and Cu^2+^) [56,57], zinc (Zn^2+^) [58,59], gallium (Ga^3^^+^) [60,61], cerium (Ce^3+^ and Ce^4^^+^) [62,63], silver (Ag^+^) [64,65,66,67], and magnesium (Mg^2+^) [68].

**Figure 2:**
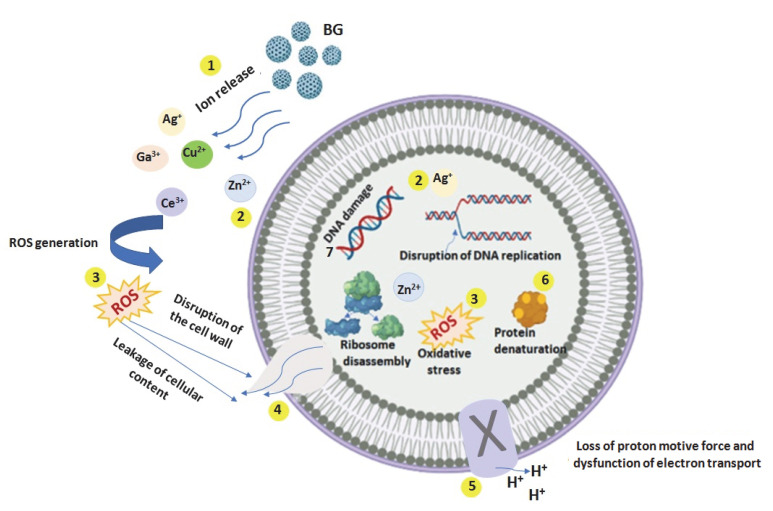
Graphic representation of the antibacterial mechanism of metal ions (created with BioRender.com (accessed on 1 September 2022). (**1**) metal ion release from BG; (**2**) direct interaction of the metal ions with the cell wall (**3**) generation of ROS (extracellular and intracellular); (**4**) elevated levels of metal ions and ROS generate the disruption of the cell wall and leaking of cellular content; (**5**) high level of ROS causes loss of the proton motive force and dysfunction of electron transport; (**6**) depending on metal ions uptake, bacterial proteins and DNA are damaged, leading to the death of the bacteria.

The antibacterial mechanism of the metal ions can be explained by the production of ROS (reactive oxygen species) and the interactions of these ions with the cell membrane, the biomolecules at the cell membrane, or the cytoplasm matrix, respectively (Figure 2) [69]:

(1) Release of the metal ions from the BGs;
(2) Direct interaction of the metal ions with the cell wall through electrostatic interactions, compromising the membrane function and hindering nutrient assimilation;
(3) Reactive oxygen species (ROS) generation, extracellular and intracellular, and oxidative stress cause damage to the proteins and DNA. Oxidative stress determined by ROS is crucial in the antibacterial effect of metal ions;
(4) The high level of metal ions attached to the cell membranes and the high ROS levels can generate the disruption of the cellular wall, and hence the leaking of the cellular content;
(5) A high level of ROS induces loss of the proton motive force and dysfunction of electron transport;
(6) Depending on metal ions uptake, these can interfere with both proteins and DNA, destruction their function, and interrupt cellular metabolism, besides the metal ions mediated ROS production [70]. The production of ROS, due to the incomplete reduction of oxygen molecules, is often reported in bacterial cells treated with metal ions. ROS are oxygen-containing derivatives composed of highly unstable oxygen radicals, such as superoxide (O_2_^−^), hydroxyl (OH^−^), hydrogen peroxide (H_2_O_2_), and singlet oxygen (O_2_) [70]. When the ratio of the generated ROS to antioxidant defenses is perturbed, the ROS concentration continuously increases and causes damage to bacterial proteins and DNA, accumulating oxidative stress and leading to a change in their functionality and the death of the bacteria [70].

The chemistry of metal ion-mediated ROS production can be systematized as follows: (i) redox-active metal ions that take part in reduction reactions by gaining electrons or oxidation reactions by losing electrons (i.e., Cu^2+^); (ii) some metal ions (i.e., Cu^2+^ and Ag^+^) can target the proteins that contain [4Fe–4S] clusters, such as the bacterial-type ferredoxins, and consequently impede their ability to transfer electrons in the metabolic reactions [71], and, as a result, Fe may be uncontrollably released into the cytoplasm, where it produces ROS; (iii) metal ions, such as Cu^2+^ and Ag^+^, may also contribute to oxidative stress in bacteria by depleting the antioxidant reserve, and, as a consequence, the cell’s antioxidative defense is compromised, making it more susceptible to subsequent metal ion-mediated ROS [72].

The generation of ROS and, thus, the toxicity displayed by the materials, can be influenced by several characteristics, such as solubility, shape, size, oxidation status, and surface area [73].

## 4. Metal Ions Incorporated Bioactive Glasses with Antibiofilm Efficiency

In recent years, several studies have demonstrated the effective antibiofilm activity of different formulations of BGs. Ag^+^, Cu^2+/+^, Ga^3+^, Ti^4+^, and Zn^2^^+^ are some examples of ions that can be used in doped BGs to target the inhibition and disruption of bacterial biofilm [47,48,50,53].

A recent study [23] reported that F18 glass, synthesized by the melting method, belonging to the SiO_2_-Na_2_O-K_2_O-MgO-CaO-P_2_O_5_ system, is a promising material for preventing and controlling bacterial biofilm. The bacterial strains *S. aureus* and *methicillin-resistant S. aureus (MRSA)* were used to evaluate the reduction of the biofilm. The results showed the inhibition of the S. aureus biofilm following direct contact with the F18 BG particles for 6 h. A reduction of the viable bacterial population by about six logs was observed. Additionally, there was a reduction in the *MRSA S. aureus* biofilm and *S. aureus* by approximately five logs when the bacterial cells were exposed to an intermediate dosage of 12 mg/mL (F18 dissolution products and powder) [23].

One of the most efficient ions against bacterial biofilm is zinc (Zn) [74,75]. Zn is a multifunctional therapeutic ion. Zn can stimulate bone formation because it is a cofactor in many enzymes and is involved in DNA replication [74]. Zn can also leak from the BG, causing oxidative stress in the intracellular medium or damage to cell membranes [76].

Zn-containing BGs have been shown to have antimicrobial properties against *S. aureus* and *E. coli* in the planktonic form [77]. 

In their investigation, Esfahanizadeh et al. [48] discovered that BG doped with 5 mol % of Zn significantly reduced the ability of Gram-negative anaerobic periodontal pathogens such as *P. gingivalis, A. actinomycetemcomitans,* and *P. intermedia* to develop a biofilm.

Zn (2.39 wt. %)-doped nano-BG particles (55SiO_2_-40CaO-5P_2_O_5_ mol %) were obtained by Paramita et al. [49] using the sol–gel method. The results showed that Zn-doped BG had better antibiofilm activity than undoped BG against the most common biofilm-forming strains: *A. aceti*, *P. aeruginosa*, and *S. aureus*. The assay was carried out at concentrations ranging from 0.1 to 1 mg/mL. At the concentration of 0.5 mg/mL, the *A. aceti* biofilm was reduced by 50%, whereas the *P. aeruginosa* and *S. aureus* biofilms were reduced by 30–40%. At selected concentrations, no cytotoxic effect was observed.

Silver (Ag) exhibits powerful antibacterial activity against a range of bacterial species [78,79,80], including antibiotic-resistant strains [81]. Incorporating Ag into BG has been proposed as a possible alternative to antibiotics for reducing infection in clinical applications [51,82,83]. Wilkinson et al. [51] aimed to elucidate the antibiofilm efficacy of Ag-doped BG (TheraGlass^TM^) against the opportunistic pathogens *P. aeruginosa* and *S. aureus*. Recently marketed for bone tissue regeneration, TheraGlass (TheraGlass^®^, MedCell, Burgess Hill, UK) is a novel, highly bioactive glass synthesized by the sol–gel method (70SiO_2_-30CaO mol%) [84]. The study [51] revealed that the treatment alone with Ag-doped BG led to a significant reduction of viable biofilm bacteria (*P. aeruginosa* and *S. aureus*). The obtained data demonstrate that Ag-doped BG is effective against established ex vivo biofilms. 

In a recent publication [52], the antibacterial activity of Ag-doped borate glasses with compositions of 60B_2_O_3_-36CaO-(4-x)P_2_O_5_-(x)Ag_2_O, where x = 0.0, 0.3, 0.5, and 1 (mol %), was examined in vitro.The dose-dependent antibacterial activity of the Ag-doped BG was demonstrated against *P. aeruginosa* preformed biofilms, with up to a 99.7% reduction in the bacterial cell counts.

Additionally, several studies [85,86] proved that Ag has an antimicrobial activity only against bacteria and fungus, but not against epithelial cells, indicating its clinical use without adverse side effects on human health.

Fan et al. [87] showed that Ag-containing MBGs (molar ratio Si:Ca:P = 80:15:5) presented an antibacterial activity against *E. faecalis* biofilm in the root canal of human teeth.

Copper (Cu) has also been used as a therapeutic ion due to its biocidal action against Gram-positive and Gram-negative bacteria and its low cytotoxicity to human cells. It is a necessary element for both human and animal existence [88]. Additionally, by enhancing angiogenesis and promoting osteogenesis, it plays a significant role in the metabolism of bone formation [88]. Similar to Ag-containing BG, the effect of Cu-containing BG against bacteria is associated with Cu ions released from the glass matrix. The antibacterial activity of Cu is achieved either directly by interacting with biomolecules or indirectly by activating oxygen species [89]. However, because bacteria are more sensitive to Ag ions than to Cu ions, the lower concentration of Ag ions has the same effect as Cu ions [89].

In 2008, the United States Environmental Protection Agency classified Cu as a metallic antimicrobial agent against many disease-causing bacteria [90].

Using poly(styrene)-block-poly(acrylic acid) (PS-b-PAA) and hexadecyltrimethylammonium bromide (CTAB) as structure-directing agents, Holquin et al. [91] produced Cu-doped hallo BG nanoparticles with the following composition: 79.5SiO_2_-(18-x)CaO-2.5P_2_O_5_-xCuO (x = 0, 2.5 or 5 mol % of CuO).

Their study showed that the composition with a greater amount of Cu was able to degrade the biofilm formed by *S. aureus* at a minimal concentration, indicating its suitability as a bactericide agent [91].

Furthermore, Bari et al. [53], demonstrated that Cu-containing MBG (2 mol %) prepared by an ultrasound-assisted one-pot synthesis inhibited bacterial growth and was also able to restrain the formation of a biofilm produced by *S. epidermidis*, and even favored its dispersion. It is known that *S. epidermidis* produces considerable quantities of polysaccharide intercellular adhesions that induce biofilm formation [92]. The findings are consistent with other studies [93,94], providing evidence of copper’s antimicrobial efficiency against bacterial growth and the development of biofilm being an effective alternative for conventional systemic therapies based on antibiotics.

Gallium (Ga) is another interesting element to be used as a therapeutic ion due to its broad-spectrum activity and immunity to the conventional resistance mechanism of bacteria associated with antibiotics. All these characteristics are linked with gallium’s pathway in bacteria metabolism. Ga acts as a “Trojan horse”, disrupting the bacterial Fe metabolism. Since the ionic radii of Ga^3+^ and Fe^3+^ are nearly identical, many biologic systems cannot discriminate between them [95]. Most bacteria need Fe^3+^ in their intracellular environment, which is reduced to Fe^2+^ during the process of many proteins. Ga can replace Fe in the cytoplasm but, unlike Fe^3+^, Ga^3+^ cannot be reduced to Ga^2+^, and the proteins become inactive [96,97]. Regarding Ga-containing BGs, gallium has been doped into both sol–gel and melt-quenching-derived glasses, and both have shown adequate efficiency against bacteria found in biomaterial-related infections [98,99,100,101]. Yazdi et al. [102] synthesized a Ga-doped zinc borate bioactive glass manifesting a sustained and controlled release of ions for at least 28 days, which was consistent with the bacteriostatic activity against *P. aeruginosa*. 

According to the study [47], melt-derived Ag- and Ga-doped phosphate glass with the following composition 10CaO-37Na_2_O-45P_2_O_5_-3Ga_2_O_3_-5Ag_2_O (mol %) contributed to the biofilm growth inhibitory effect on *P. aeruginosa* (up to 2.68 reductions in log10 values of the viable counts compared with controls). The composition may offer a successful choice to combat opportunistic pathogens such as *P. aeruginosa*-associated infections due to the controlled release of antibacterial Ga and Ag ions at the site of infection. The melt-derived Ag- and Ga-doped phosphate glass was also tested in terms of the inhibition of biofilm formation against *P. gingivalis* and *S. gordonii* periodontal pathogens [103]. The glass developed in the study [103] reduced biofilm formation of *P. gingivalis* after 7 days of exposure by combining the actions of Ga and Ag synergistically. Ag ions destabilize the biofilm matrix to increase the biofilm’s contact area, hence enhancing the chances of the Ag and Ga ions subsequently killing the bacteria.

Recently, Tellurium (Te), an element from the chalcogens group that exhibits several oxidation states, has been studied for biological applications due to its antimicrobial, antioxidant, and antitumoral properties [54,104,105,106]. Telluride (Te^2−^)→elemental tellurium (Te^0^)→tellurite (TeO_3_^2^^−^)→tellurate (TeO_4_^2−^) are only a few of the redox states in which Tellurium can be found. The tellurite (TeO_3_^2−^) is highly toxic for most bacteria (e.g., *Escherichia coli*) even at concentrations of 1 μg mL^−1^ [107].

This opens a new perspective for the Te element in terms of biomaterials to prevent bacterial infections in tissue engineering applications.

A recent study [54] analyzed the antibacterial activity against bacterial biofilm of Te-doped BGs. The investigated Te-doped BG showed a significant biofilm metabolic reduction for both *S. aureus* and *S. epidermidis*, the most frequent strains involved in orthopedics infections [108].

The antibacterial activity of Te is due to a combination of different mechanisms that are likely not yet fully disclosed. Turner et al. [109] gave an explanation of some events. Briefly, tellurite can exceed the outer membrane due to the environmental pH variation and then exert its toxic action in the cytoplasmic compartment by triggering an increase in the generation of reactive oxygen species (ROS) [110]. The tellurite influences the activity of the superoxide dismutase (SOD) that is necessary to counteract the oxygen species (ROS) formed. 

## 5. Conclusions and Future Perspectives

Bone and joint infections during orthopedic surgery due to bacterial biofilm on prosthetic implants is a growing public health concern. Bacterial biofilms are among the most critical problems currently confronting medicine due to their growing antibiotic resistance and unique defense mechanisms. The development of multifunctional biomaterials for bone regeneration with good drug delivery capabilities that exhibit antibacterial activity against bacterial biofilm represents the current focus in tissue engineering research. BGs show considerable potential for regenerative medicine because they can have simultaneous antibacterial, antibiofilm, and regenerative properties.

Studies have emphasized the antibacterial properties of BGs, frequently highlighting how their effectiveness is related to their particle size or, if they are doped with biocide metal ions, how they can damage the cell membrane of bacteria or generate ROS. F18 BG is a promising material showing a reduction of *S. aureus* and *MRSA* biofilms following direct contact. Comparing the efficacy of 45S5 and S53P4 BGs against the biofilm generated by *MRSA* and *V. parvula*, it was found that 45S5 BG exhibited a higher reduction of the biofilm than S53P4 BG, and the particle size played a significant role in the effectiveness against biofilms, with the smaller particles being more effective. 

The incorporation of biocide metal ions such as Ag, Cu, Zn, or Ga into BGs demonstrated more promising antibiofilm effects than the undoped BGs. Ag-doped BG, TheraGlass^TM^ (TheraGlass^®^, MedCell, Burgess Hill, UK), recently marketed for bone tissue regeneration, showed a notable reduction of *P. aeruginosa* and *S. aureus* biofilms. Among the doped BGs, Te-doped BG showed a significant biofilm reduction of the most frequent strains involved in orthopedic infections, namely *S. aureus* and *S. epidermidis*. 

Despite the significant progress in the search for efficient BG compositions for the cure of mature biofilms, only a few have been evaluated for in vivo trials.

In the future, considerable research needs to be devoted to developing BGs that will trickle from the study to clinical applications, thus enabling a reduction in biofilm infections in orthopedic surgery and other medical specialties.

## Figures and Tables

**Table 1:** The inhibitory ability of bacterial biofilm of some metal ions.

Ions	BG Composition	Synthesis	Microbial Biofilm	Ref.
**Ga^3+^** **+Ag^+^**	10CaO-37Na_2_O-45P_2_O_5_-3Ga_2_O_3_-5Ag_2_O (mol %)	Melting	Inhibition of *P. aeruginosa* biofilm	[47]
**Zn^2+^**	Zn doped BG (5 mol %)	Sol–gel	Reduced biofilm formation of *A. actinomycetemcomitans, P. gingivalis,* and *P. intermedia*	[48,49]
	55SiO_2_-40CaO-5P_2_O_5_ (mol %) Zn (2.39 wt. %)	Sol–gel	Inhibition of *S. aureus, P. aeruginosa*, and *A. aceti* biofilms	
**Ti^4+^**	40P_2_O_5_⋅16CaO⋅24MgO⋅17.5NaO⋅2.5TiO_2_ (mol %)	Melting	Inhibitory effect on *S. mutans* biofilm	[50]
**Ag^+^**	70SiO_2_-28CaO-2AgO (mol %)	Sol–gel	*S. aureus* and *P. aeruginosa* biofilms formation was entirely inhibited	[51,52]
	60B_2_O_3_–36CaO–(4–X)P_2_O_5_–(X)Ag_2_O x = 0.3, 0.5, 1 (mol %)	Sol–gel	Eradicated *P. aeruginosa* biofilm by up to 99.7%.	
**Cu^2+^**	Cu (2 mol %)-doped MBGs	Sol–gel	Disrupted the biofilm matrix of *S. epidermidis*	[53]
**Te^4+^**	48.6-xSiO_2_-16.7Na_2_O-34.2CaO-0.5P_2_O_5_-xTeO_2_ x = 1, 5 (mol %)	Melting	Ability to inhibit *S. aureus* and *S. epidermidis* biofilms formation	[54]
